# Hematopoietic Stem Cell Transplantation for Acute Myeloid Leukemia: An Overview of Systematic Reviews

**DOI:** 10.1155/2022/1828223

**Published:** 2022-10-07

**Authors:** Peijie He, Juan Liang, Wanjun Zhang, Shengyun Lin, Hanting Wu, Qiushuang Li, Xiujuan Xu, Conghua Ji

**Affiliations:** ^1^School of Public Health, Zhejiang Chinese Medical University, Hangzhou 310053, Binwen Road 548#, China; ^2^Department of Hematology, The First Affiliated Hospital of Zhejiang Chinese Medical University, Hangzhou 310006, Youdian Road 46#, China; ^3^Clinical Evaluation Center, The First Affiliated Hospital of Zhejiang Chinese Medical University, Hangzhou 310006, Youdian Road 46#, China; ^4^Critical Care Department, Tongde Hospital of Zhejiang Province, Hangzhou, China

## Abstract

**Background:**

Hematopoietic stem cell transplantation (HSCT) has become the main treatment for acute myeloid leukemia (AML) and has been studied in many systematic reviews (SRs), but strong conclusions have not been drawn yet.

**Objective:**

This study aimed to summarize and critically evaluate the methodological and evidence quality of SRs and meta-analysis on this topic.

**Methods:**

PubMed, Embase, the Cochrane Library, and Web of Science were searched for SRs/meta-analyses regarding HSCT for AML. Two reviewers assessed the quality of SRs/meta-analyses in line with AMSTAR-2 and evaluated the strength of evidence quality with the grading of the evaluation system (GRADE) for concerned outcomes independently.

**Results:**

12 SR/Meta articles were included, and the AMSTAR-2 scale showed that the quality grade of all articles was low or very low. GRADE results showed 29 outcomes, 2 of which were high, 12 were moderate, and 15 were low. Limitations and inconsistency were the most important factors leading to degradation, followed by imprecision and publication bias. Allo-SCT had better OS and DFS benefits than auto-SCT and significantly reduced the relapse in intermediate-risk AML/CR1 patients. Auto-SCT was associated with lower TRM than allo-SCT but generally had higher relapse. The results should be confirmed further for the low or moderate evidence quality.

**Conclusion:**

Current SRs show that allo-SCT in the treatment of AML might improve the OS, RFS, and DFS. Auto-SCT has significantly lower TRM but higher RR. Whether bone marrow transplantation is superior to nonmyeloablative chemotherapy remains to be evaluated. Meanwhile, the quality of methodology needs to be further improved. The intensity of evidence was uneven, and the high-quality evidence of outcomes was lacking. Considering the limitations of our overview, more rigorous and scientific studies are needed to fully explore the efficacy of different interventions of HSCT in AML, and clinicians should be more cautious in the treatment.

## 1. Introduction

Acute myeloid leukemia (AML) is a hematopoietic stem cell malignancy with high heterogeneity [1]. It is mainly characterized by clonal expansion of myeloid primordial cells in peripheral blood, the bone marrow, and/or other tissues [2]. AML is the most common acute leukemia in adults, with an average annual incidence of over 20000 cases in the United States [3]. It accounts for one-third of leukemia cases diagnosed in the United States annually, and its mortality is also the highest among leukemia cases [4]. AML is distinguished from acute lymphoblastic leukemia by cytochemical staining and morphology, and AML is divided into six categories according to genetics and clinical manifestations [4, 5]. Most AMLs occur in the bone marrow and peripheral blood [6]. Clinical manifestations of AML include signs of leukocytosis and bone marrow failures, such as anemia and thrombocytopenia, followed by infection, bleeding, or diffuse intravascular coagulation [3, 7]. It is necessary for patients with suspected AML to undergo bone marrow examination, and determine the diagnosis and analyze AML subtypes through cytogenetics and molecular examination, determine treatment, and evaluate prognosis [8–10].

The incidence rate of AML is increasing. The average age of patients diagnosed with AML is 65–70 years [11]. In all age groups, the incidence rate of AML in men is higher than that in women [12]. Its incidence is age-related, and it increases with health status changes with age. Although the treatment and prognosis of AML have improved, it is only applicable to young patients [3, 13]. The prognosis of most elderly patients is still significantly poor, with more complications, such as hypertension, chronic obstructive pulmonary disease, diabetes, heart disease, and kidney and other organ dysfunctions [11]. Thus, it is impossible to tolerate intensive chemotherapy, and 70% of patients aged 65 years and older die within 1 year after diagnosis. Thus, they face higher treatment-related mortality (TRM) [3]. In contrast, individuals over the age of 65 years are more likely to have adverse cytogenetic risk characteristics. They are insensitive to chemotherapy, frequently exhibit multidrug resistance, and are vulnerable to treatment-related toxicity [11, 13]. Therefore, the optimal treatment for elderly patients with AML has not yet been established. AML is the second most common type of acute leukemia in children [14]. Although the treatment and prognosis of pediatric AML have improved, the overall survival (OS) rate is still <70% [15, 16]. At present, the traditional treatment is the “3–7” standard regimen; that is, cytarabine and daunorubicin or Idamycin are taken continuously for 7 and 3 days, respectively [17].

AML treatment mainly involves induction therapy to remission, followed by consolidation treatment. Induction therapy aims to achieve complete remission (CR), preferably without measurable residual disease [18]. Studies have shown that patients who achieve CR have better survival rates. The two common induction therapies for AML include (1) cytotoxic chemotherapy and (2) demethylated drugs [4]. The goals of postremission treatment are to prevent relapse and to achieve timely consolidation treatment to eradicate residual diseases. Options available for consolidation include cytotoxic chemotherapy (e.g., cytarabine) and hematopoietic stem cell transplantation (HSCT) [4, 19]. The choice of treatment is dependent on the patient's characteristics.

HSCT can be considered the most successful treatment for AML, and it is an alternative to conventional chemotherapy. It provides moderate- or high-risk patients after remission with survival advantages, reduces the recurrence rate, and treats relapsed AML. However, it is also associated with high transplant-related mortality, graft-versus-host disease, and some late sequelae. Thus, the advantages and disadvantages of HSCT should be carefully considered [20].

More patients are undergoing HSCT for the treatment of AML. Autologous HSCT has rarely been used in recent years, but it has no donor source limitation and low transplantation-related mortality and results in an improved quality of life after transplantation [21]. However, relapse is the primary cause of treatment failure. More than half of patients with AML relapse after transplantation, and their prognosis is usually poor [22]. In addition, even with cell therapy or research drugs, only a small number of patients can be saved in the long term.

Over the years, several systematic reviews (SRs)/meta-analyses of HSCT for the treatment of AML have been published, but the methodological and evidence qualities of the outcomes remain to be evaluated. Therefore, we conducted an overview using the A MeaSurement Tool to Assess Systematic Reviews (AMSTAR)-2 scale and GRADE system to summarize and critically evaluate the SRs/meta-analyses of HSCT for the treatment of AML to provide some insights into the development of evidence-based medical guidelines and further studies for clinicians.

## 2. Methods

This method partially comprised a summary of the methods used. We used the participant, intervention, comparison, and outcome (PICO) pattern to improve the inclusion and exclusion criteria for our overview of reviews. The present work has been registered at the International Prospective Register for Systematic Reviews, identification code (CRD42022301689).

### 2.1. Inclusion and Exclusion Criteria

The inclusion criteria were as follows: (1) SRs/meta-analyses of HSCT for the treatment of AML; (2) reviews comprising patients clearly diagnosed with AML, with no restrictions on sex, age, race, occupation, disease course, disease severity, and treatment remission degree; (3) reviews with intervention measures comprising HSCT (allo-SCT, auto-SCT, autologous bone marrow transplantation (ABMT), and bone marrow transplantation (BMT)) and control measures comprising chemotherapy and nontransplantation therapy; (4) reviews reporting at least one of the following outcomes: OS, disease-free survival (DFS), event-free survival, relapse-free survival (RFS), relapse rate (RR), TRM, and second CR. The exclusion criteria were as follows: (1) duplicated literature; (2) reviews comprising patients with AML complicated by other diseases; (3) experience summary, case report, conference abstract, reviews unable to obtain full text, and other irrelevant literature.

### 2.2. Retrieval Strategy

We searched PubMed, Embase, Web of Science, and Cochrane Library databases to obtain all reviews on HSCT in the treatment of hematological diseases. We searched the PubMed and Cochrane Library databases by combining Medical Subject Heading terms with text words and searched the Embase database by combining Emtree terms with free words. In addition, we assessed the references in all known articles and SRs to obtain the relevant literature that could not be retrieved from the database search. Two reviewers (PJ He and HT Wu) independently searched the literature and resolved the differences with a third reviewer.

### 2.3. Literature Selection

Eligible SRs were independently selected by two reviewers (PJ He and HT Wu) in two steps. First, after removing duplicates using EndNote X9 software, the applicability of the title, abstract, and reference list of the obtained reviews was screened. Second, all articles that met the inclusion criteria in the first step were retrieved for a detailed full-text assessment to determine whether they were qualified.

### 2.4. Data Extraction

According to the characteristics of the included reviews, two reviewers (PJ He and HT Wu) independently extracted the following basic information from the literature: first author, publication year, the number and type of participants, intervention group, control group, and outcomes.

### 2.5. Quality Assessment (A MeaSurement Tool to Assess Systematic Reviews)

Two authors (PJ He and HT Wu) independently assessed the quality of the included SRs using AMSTAR-2 [23]. Any dispute was discussed with a third investigator. The AMSTAR-2 tool contains 16 items, 7 of which are critical items (2, 4, 7, 9, 11, 13, and 15) [24]. According to its criteria, the evaluations are “yes,” “partially yes,” and “no.” Each SR is categorized as “high quality,” “moderate quality,” “low quality,” and “critically low.”

### 2.6. GRADE Scoring

The GRADE system [25] was used to assess the evidence quality of the concerned outcomes and classified the evidence quality into four different levels: high, moderate, low, and very low. According to its usage guidelines, we mainly investigated the limitation, imprecision, inconsistency, indirectness, and publication bias. Two authors (PJ He and HT Wu) independently assessed the quality of each outcome, and ambiguities were resolved by discussion with the third coauthor.

## 3. Results

### 3.1. Study Identification

**Figure 1:**
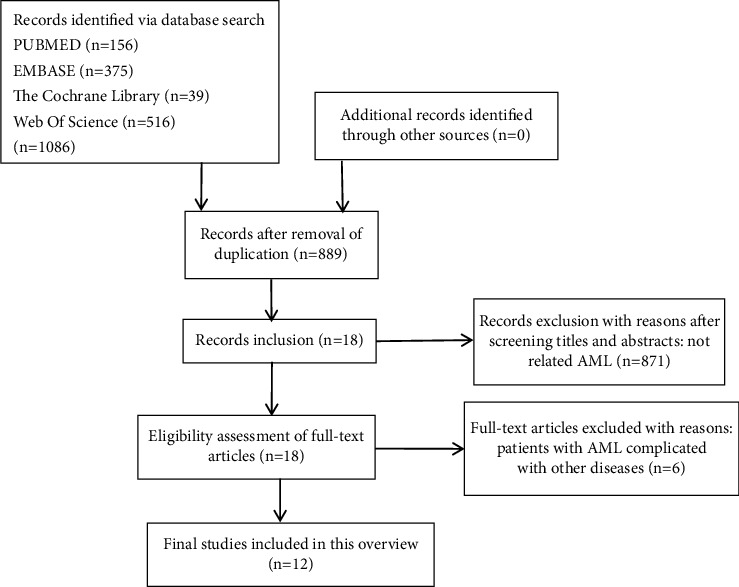
Flow diagram of study selection.

We identified 1086 literature reviews through a database search, and 197 duplicated studies were excluded. After screening titles and abstracts, 871 studies were excluded. Eighteen full-text articles were selected for further evaluation, and twelve reviews were finally included in this overview. The selection process is presented in Figure 1. The main characteristics of the included reviews are summarized in Table 1.

### 3.2. Critical Appraisal of the Included SRs

Based on AMSTAR-2, we assessed the methodological quality of the twelve SRs included in this overview. Two SRs [26, 27] were deemed to be of low quality according to AMSTAR-2. The remaining SRs were deemed to be of critically low quality. The following were considered critical items: ① all included SRs registering a protocol previously (item 2); ② most SRs using a comprehensive literature search strategy (item 4); ③ all SRs without the key factors of item 7 (a list of excluded studies); ④ two SRs [26, 27] using a satisfactory tool for assessing the risk of bias (RoB) (item 9); ⑤ eleven SRs [26–36] using appropriate methods for the statistical combination of results (item 11); ⑥ reviews with the quality of the research included in all the literature being different, with SRs accounting for RoB when interpreting/discussing the results of the review (item 13); ⑦ seven SRs [26–30, 32, 34] using statistical tests or funnel plots to investigate publication bias and discussing their possible effect on the results (item 15). The following were considered non-critical items: ① all SRs including the components of PICO and describing the included studies in adequate detail (item 1, item 8);② seven SRs [26, 29, 32, 34–37] performed study selection in duplicate (item 5);③ eight SRs[26–29, 32, 34–36]performed duplicate data extraction (item 6); ④ four SRs [29, 34, 36, 37] reported the source of funding (item 10);⑤ two SRs [26, 27] assessed the potential effect of RoB on the results of the meta-analysis (item 12); ⑥ten SRs [26–30, 32–36] provided discussion of the significant heterogeneity(item 14);⑦only four SRs[28, 31, 33, 37]reported no potential source of conflict of interest (item 16). No SRs explained the reasons for inclusion in the study designs (item 3). The quality of all the included reviews is presented in Table 2.

### 3.3. Evidence Quality of Outcomes

Meta-analysis was conducted for 12 SRs, including 29 outcomes in total. The qualities of the 2, 12, and rest of the outcomes were high, moderate, and low, respectively, according to the GRADE system. The qualities of outcomes are summarized in Table 3.

### 3.4. Meaningful Outcome Comparison

The main meaningful outcomes of the nine studies were shown to compare different interventions or populations. From the comparison of the two included studies [27, 28], it was found that allo-SCT had an OS advantage ((hazard ratio (HR), 0.84 (0.73–0.97) and HR, 0.43 (0.22–0.84), respectively), and it significantly reduced relapse in patients with intermediate-risk AML/first CR ((CR1) (HR, 0.53 (0.42–0.66) and HR, 0.58 (0.45–0.75), respectively). The HRs for RFS were 0.82 (0.73–0.92) and 0.68 (0.48–0.95) in auto-SCT and allo-SCT, respectively, indicating that allo-SCT significantly reduced the incidence of death or relapse. However, allo-SCT had a generally higher TRM rate, with HRs of 4.16 (3.37–5.15) and 3.09 (1.38–6.92) in allo-SCT and auto-SCT, respectively. By comparing the four included studies [26–28, 32], it was found that patients with AML who were not at intermediate risk were less likely to experience treatment-related deaths. Allo-SCT may also have DFS advantages in patients with FLT3/ITD AML.

From the comparison of the three included studies [30, 32, 35], it was found that allo-SCT had better OS and DFS than auto-SCT in patients with AML/CR1 (HR, 0.90 (0.82–0.97) and HR, 0.89 (0.80–0.98), respectively). Auto-SCT had higher relapse (RR, 0.79 (0.72–0.87)) and a lower survival rate from relapse (HR, 2.09 (1.41–3.08)). However, auto-SCT had a lower TRM rate during the first remission (RR, 1.90 (1.34–2.70)). There was an evident reduction in death or AML relapse with allo-SCT in CR1 (HR, 0.80 (0.74–0.86)). ABMT may not effectively reduce mortality (RR, 0.94 (0.84–1.09)), but it relatively had fewer relapses (RR, 0.85 (0.75–0.97)).

In the two included studies [34, 35], ABMT was used as an intervention treatment. ABMT may not have a better survival advantage for patients with AML, but it may reduce relapse. A comparison is presented in Table 4.

## 4. Discussion

### 4.1. Methodological Quality

AMSTAR-2 was used to assess the methodological quality of the included studies. The results showed that the overall quality of the 12 SRs was low. The main reasons for the low quality are as follows: ① all SRs did not provide the list of excluded literature; ② the reasons for the inclusion of study designs were not explained (12 SRs included randomized controlled trials and prospective cohort studies, but none of the literature explained why these types of studies were included); ③ most SRs failed to use reasonable tools to correctly and comprehensively assess the RoB included in the study; ④ the quality of the included studies in SRs was different, and publication bias was not fully investigated in the quantitative synthesis. The literature report should pay attention to the accurate description of PICO and comprehensive literature retrieval (at least an example of all electronic retrieval strategies in one database) and should describe in detail the process of literature screening, data extraction, and quality evaluation; source of funds included in the study; evaluation methods of bias, publication and selective report bias; subsequent data consolidation; and the potential effect on the results of SRs and bias.

### 4.2. Appraisal of the Quality of Evidence

The GRADE system was used to evaluate the evidence quality of the outcomes. The quality of the outcomes was uneven. High evidence quality included RR and TRM in one SR. [28]. Moderate evidence qualities included the following:① RFS and OS in two SRs [28]; ② RR in three SRs [26, 27, 36]; ③ TRM in one SR [27]; ④ survival from relapse in one SR [30]; ⑤ death or RR in two SRs [35, 36]; ⑥ death rate in one SR [36]. The rest of the outcomes were of low evidence quality. This indicates that there may be differences between the conclusion and the actual situation. The main reasons include the following: ① limitation, no distribution concealment, blind method, and loss to follow-up report; ② imprecision, the small sample size of included studies, a poor overlap degree of confidence intervals of different studies, and a wide confidence interval; ③ indirectness, a certain gap in the intervention in some studies; ④ publication bias, asymmetric funnel plots or a significantly small number of included studies; ⑤ moderate heterogeneity. The reasons for most of the degradation are limitation and inconsistency. However, the outcomes still have imprecision and publication bias.

In clinical practice, more attention should be paid to achieving high-quality outcomes. Considering the outcomes that are of moderate quality, it is reasonable to be extremely cautious when applying these to clinical decision-making. Regarding low-quality outcomes, additional studies are required to confirm this evidence. Nevertheless, we look forward to better evidence in future studies that can support further clinical development.

### 4.3. Result Interpretation

Previous studies have shown that allo-SCT has a certain treatment potential in AML, largely due to the immune-mediated graft-versus-leukemia effect, and relies on durable donor T cell engraftment. This can be achieved by intensive myeloablative conditioning, which has a lower RR but is related to an increase in TRM [38]. Most importantly, we revealed that allo-SCT probably provided more significant OS and DFS benefits than auto-SCT for patients with AML in CR1. Moreover, it showed a higher TRM rate after allo-SCT and superior OS and DFS [39]. In this overview, auto-SCT might have a potentially high risk of relapse, and we found results similar to those reported in this article. Some treatments fail to eliminate all malignant cells, such as auto-SCT, resulting in relapse or even death [40]. It has been mentioned in this overview that ABMT is similar to nonmyeloablative chemotherapy in terms of OS with no significant advantage. A few results do not support the routine use of ABMT in adult patients with AML in CR1 [34]. A previous study [41] showed that some genetic biomarkers (IGF2R, CTSA, and ATP6AP2) can subdivide AML patients into different prognosis groups. If gene biomarkers can be used for personalized treatment, we could take the corresponding therapeutic schedule. Future investigations of HSCT in AML should focus on minimizing the TRM rate and reducing the risk of disease recurrence. The main challenge is to further increase the survival rate while optimizing the quality of life of all patients.

### 4.4. Limitations

Our analysis has some limitations which are as follows: (1) the grading process of evidence quality in the GRADE system is subjective, and there may be some differences among different researchers; (2) the outcomes selected by different studies are different; thus, their evaluation will affect the result comparison and conclusion analysis; (3) there may be publication bias, which reduces the credibility of this study; (4) there may be heterogeneity, mainly due to the great differences in the included studies. Therefore, it is necessary to further clarify the inclusion and exclusion criteria and perform an appropriate subgroup analysis to overcome these limitations.

## 5. Conclusions

This overview found that allo-SCT in the treatment of AML might improve OS, RFS, and DFS. Auto-SCT may have a significantly lower TRM but higher RR than allo-SCT. Whether bone marrow transplantation is superior to nonmyeloablative chemotherapy remains unclear. Moreover, patients with AML who are at intermediate or high risk are likely to experience treatment-related deaths. HSCT for the treatment of AML has certain advantages over the traditional method, but the methodological quality of SRs needs to be further improved. The intensity of the evidence is uneven, and there is significantly little evidence. Considering the limitations of our overview, more rigorous and scientific studies are required to fully explore the efficacy of HSCT in AML, with clinicians being more cautious in the treatment.

## Figures and Tables

**Table 1:** Characteristics of included reviews.

Author (y)	Number of included articles	Number of patients	Type of patients	Intervention	Comparison	Outcome
Hoffman (2021) [36]	12	1928	Children or adolescents under the age of 21 with the first relapse of AML	Allo-SCT, auto-SCT, undefined type of SCT	Chemotherapy	abcd
Li (2019) [27]	11	1133	Patients with intermediate-risk AML in CR1	Auto-SCT	Allo-SCT	befg
Rashidi (2016) [28]	13	749	>60 years AML patients	Allo-SCT	Further chemotherapy or no treatment	Be (6 months, 1, 2, 3 years)
Ma (2015) [25]	9	772	FLT3/ITD AML patients	Allo-SCT	Auto-SCT, chemotherapy	bcg
Li (2015) [26]	9	1950	Patients with intermediate-risk AML in CR1	Allo-SCT	Nonallo-SCT (auto-SCT/chemotherapy)	befg
Wang (2010) [29]	13	3027	AML patients in CR1	Auto-SCT	Further chemotherapy or no treatment	bcfg
Krauter (2009) [30]	8	180	<60 years old with AML patients and reciprocal translocations involving chromosome band 11q23 [t (11q23)]	Allo-SCT	Chemotherapy or auto-SCT	be
Koreth (2009) [31]	24	6007	AML patients in CR1	Allo-SCT	Nonallo-SCT (auto-SCT/chemotherapy)	be
Schaich (2007) [32]	8	131	AML patients (median age 50 (18–60) years) exhibiting trisomy8 within a noncomplex karyotype	High-dose cytarabine + allo-SCT/auto-SCT	High-dose cytarabine	be
Nathan (2004) [33]	6	1044	Adult patients with AML	Autologous bone marrow transplantation (ABMT)	Nonmyeloablative chemotherapy or no further treatment	bc
Levi (2004) [34]	6	4110	AML patients in CR1	ABMT	Chemotherapy	bd
Bleakley (2002) [35]	10	2200	Pediatric AML patients in CR1	ABMT	BMT(with a matched sibling donor)/nonmyeloablative chemotherapy	bcdfg

a: CR2, second complete remission; b: OS, overall survival; c: DFS, disease-free survival; d: EFS, event-free survival; e: RFS, relapse-free survival; f: TRM, treatment-related mortality; g: RR, relapse rate.

**Table 2:** Quality of the included reviews as rated on the AMSTAR-2 scale.

Author (Y)	AMSTAR-2 criteria																Overall quality
	1	2	3	4	5	6	7	8	9	10	11	12	13	14	15	16	
Hoffman (2021) [36]	Y	Y	N	Y	Y	N	N	Y	N	Y	N	N	Y	N	N	Y	CL
Li(2019) [27]	Y	Y	N	Y	N	Y	N	Y	N	N	Y	N	Y	Y	Y	Y	CL
Rashidi(2016) [28]	Y	Y	N	Y	Y	Y	N	Y	N	Y	Y	N	Y	Y	Y	N	CL
Ma (2015) [25]	Y	Y	N	Y	Y	Y	N	Y	Y	N	Y	Y	Y	Y	Y	N	L
Li (2015) [26]	Y	Y	N	Y	N	Y	N	Y	Y	N	Y	Y	Y	Y	Y	N	L
Wang (2010) [29]	Y	Y	Y	Y	N	N	N	Y	N	N	Y	N	Y	Y	Y	N	CL
Krauter (2009) [30]	Y	Y	N	N	N	N	N	Y	N	N	Y	N	Y	N	N	Y	CL
Koreth (2009) [31]	Y	Y	Y	Y	Y	Y	N	Y	N	N	Y	N	Y	Y	Y	N	CL
Schaich (2007) [32]	Y	Y	N	N	N	N	N	Y	N	N	Y	N	Y	Y	N	Y	CL
Nathan (2004) [33]	Y	Y	Y	Y	Y	Y	N	Y	N	Y	Y	N	Y	Y	Y	N	CL
Levi (2004) [34]	Y	Y	Y	Y	Y	Y	N	Y	N	N	Y	N	Y	Y	N	N	CL
Bleakley (2002) [35]	Y	Y	N	Y	Y	Y	N	Y	N	Y	Y	N	Y	Y	N	N	CL

Y: yes; PY: partial yes; N: no; H: high; M: moderate; L: low; CL: critically low. 1: Did the research questions and inclusion criteria for the review include the components of PICO? 2: Did the report of the review contain an explicit statement that the review methods were established prior to the conduct of the review and did the report justify any significant deviations from the protocol? 3: Did the review authors explain their selection of the study designs for inclusion in the review? 4: Did the review authors use a comprehensive literature search strategy? 5: Did the review authors perform study selection in duplicate? 6: Did the review authors perform data extraction in duplicate? 7: Did the review authors provide a list of excluded studies and justify the exclusions? 8: Did the review authors describe the included studies in adequate detail? 9: Did the review authors use a satisfactory technique for assessing the risk of bias (RoB) in individual studies that were included in the review? 10: Did the review authors report on the sources of funding for the studies included in the review? 11: If meta-analysis was performed, did the review authors use appropriate methods for statistical combination of results? 12: If meta-analysis was performed, did the review authors assess the potential impact of RoB in individual studies on the results of the meta-analysis or other evidence synthesis? 13: Did the review authors account for RoB in primary studies when interpreting/discussing the results of the review? 14: Did the review authors provide a satisfactory explanation for, and discussion of, any heterogeneity observed in the results of the review? 15: If they performed quantitative synthesis did the review authors carry out an adequate investigation of publication bias (small study bias) and discuss its likely impact on the results of the review? 16: Did the review authors report any potential sources of conflict of interest, including any funding they received for conducting the review.

**Table 3:** Quality of evidence in included reviews with GRADE.

Author (Y)	Intervention (treatment group vs control group)	Outcomes (n)	Risk of bias	Inconsistency	Indirectness	Imprecision	Publication bias	Quality of evidence
Hoffman (2021) [36]	SCT (allo-SCT, auto-SCT, undefined type of SCT) vs chemotherapy	CR2 (12)	0	−1	0	0	0	M
		OS (12)	0	−1	0	0	0	M
Li (2019) [27]	Auto-SCT vs allo-SCT	RFS (10)	0	−1	0	0	0	M
		OS (10)	0	−1	0	0	0	M
		RR (7)	0	0	0	0	0	H
		TRM (4)	0	0	0	0	0	H
Rashidi (2016) [28]	Allo-SCT vs further chemotherapy or no treatment	RFS (10)	0	0	0	−1	0	M
		OS (12)	0	0	0	−1	0	M
Ma (2015) [25]	Allo-SCT/auto-SCT vs chemotherapy; allo-SCT vs auto-SCT	OS (9)	−1	−1	0	0	0	L
		RR (4)	−1	0	0	0	0	M
		DFS (5)	−1	−1	0	0	0	L
Li (2015) [26]	Allo-SCT vs auto-SCT or (and) chemotherapy	RFS (6)	−1	−1	0	0	0	L
		RR (8)	−1	0	0	0	0	M
		TRM (4)	−1	0	0	0	0	M
		OS (4)	−1	−1	0	0	0	L
Jing (2010) [29]	Auto-SCT vs further chemotherapy or no treatment	OS (11)	−1	−1	0	0	0	L
		RR (11)	−1	−1	0	0	0	L
		DFS (13)	−1	−1	0	0	0	L
		TRM (11)	−1	−1	0	0	0	L
		Survival from relapse (3)	−1	0	0	0	0	M
Krauter (2009) [30]	Allo-SCT vs auto-SCT or (and) chemotherapy	OS	0	0	0	0	0	H
		RFS	0	0	0	0	0	H
Koreth (2009) [31]	Allo-SCT vs auto-SCT or (and) chemotherapy	OS (15)	−1	−1	0	0	0	L
		RFS (18)	−1	−1	0	0	0	L
Schaich (2007) [32]	High-dose cytarabine + allo-SCT/auto-SCT vs high-dose cytarabine	OS	0	0	0	0	0	H
		RFS	0	0	0	0	0	H
Nathan (2004) [33]	ABMT vs nonmyeloablative chemotherapy or no further treatment	OS (5)	−1	−1	0	0	−1	L
		TRM (6)	−1	0	0	0	−1	L
		DFS (6)	−1	0	0	0	−1	L
Levi (2004) [34]	ABMT vs chemotherapy	Death rate (6)	0	−1	0	0	−1	L
		Death or relapse rate(6)	0	0	0	0	−1	M
Bleakley (2002) [35]	ABMT vs BMT (MSD)/ABMT vs nonmyeloablative chemotherapy	RR (3)	0	0	0	0	−1	M
		Death rate (3)	0	0	0	0	−1	M
		Death or relapse rate (4)	0	0	0	0	−1	M
		TRM (3)	0	−1	0	0	−1	L

−1:serious(downgrade by one level); −2: extremely serious (downgrade by two levels); 0: not serious; H: high; M: moderate; L: low; CL: critically low CR2, second complete remission; OS, overall survival; DFS, disease-free survival; EFS, event-free survival; RFS, relapse-free survival; TRM, treatment-related mortality; RR, relapse rate.

**Table 4:** Main meaningful outcome comparison in included reviews.

Author (y)	Type of patients	Intervention	Outcome(s)							
			OS	DFS	RFS	TRM	RR	Survival from relapse	Death rate	Relapse or death rate
Li (2019) [27]	Patients with intermediate-risk AML in CR1	Allo-SCT	HR = 0.84(0.73–0.97)		HR = 0.82(0.73–0.92)	HR = 4.16(3.37–5.15)	HR = 0.53(0.42–0.66)			
Li (2015) [26]	Patients with intermediate-risk AML in CR1	Allo-SCT	HR = 0.43(0.22–0.84)		HR = 0.68(0.48–0.95)	HR = 3.09(1.38–6.92)	HR = 0.58(0.45–0.75)			
Ma (2015) [25]	FLT3/ITD AML patients	Allo-SCT	OR = 2.88(2.04–4.05)	OR = 2.84(1.89–4.25)			OR = 0.09(0.05–0.18)			
Koreth (2009) [31]	AML patients in CR1	Allo-SCT	HR = 0.90(0.82–0.97)		HR = 0.80(0.74–0.86)					
Jing (2010) [29]	AML patients in CR1	Allo-SCT	HR = 1.05(0.91–1.21)	HR = 0.89(0.80–0.98)		RR = 1.90(1.34–2.70)	RR = 0.79(0.72–0.87)	HR = 2.09(1.41–3.08)		
Koreth (2009) [31]	AML patients in CR1	Allo-SCT	HR = 0.90(0.82–0.97)		HR = 0.80(0.74–0.86)					
Levi (2004) [34]	AML patients in CR1	ABMT							RR = 0.94(0.84–1.09)	RR = 0.85(0.75–0.97)
Nathan (2004) [33]	Adult patients with AML	ABMT	1.01(0.89–1.15)	1.24(1.06–1.44)		OR = 2.63(1.60–4.32)				
Levi (2004) [34]	AML patients in CR1	ABMT						RR = 0.94(0.84–1.09)	RR = 0.85(0.75–0.97)	

## Data Availability

The data supporting the current study are given in the article.
